# Structure Prediction of RNA Loops with a Probabilistic Approach

**DOI:** 10.1371/journal.pcbi.1005032

**Published:** 2016-08-05

**Authors:** Jun Li, Jian Zhang, Jun Wang, Wenfei Li, Wei Wang

**Affiliations:** National Laboratory of Solid State Microstructures, School of Physics, Collaborative Innovation Center of Advanced Microstructures, Nanjing University, Nanjing, China; University of Missouri, UNITED STATES

## Abstract

The knowledge of the tertiary structure of RNA loops is important for understanding their functions. In this work we develop an efficient approach named RNApps, specifically designed for predicting the tertiary structure of RNA loops, including hairpin loops, internal loops, and multi-way junction loops. It includes a probabilistic coarse-grained RNA model, an all-atom statistical energy function, a sequential Monte Carlo growth algorithm, and a simulated annealing procedure. The approach is tested with a dataset including nine RNA loops, a 23S ribosomal RNA, and a large dataset containing 876 RNAs. The performance is evaluated and compared with a homology modeling based predictor and an *ab initio* predictor. It is found that RNApps has comparable performance with the former one and outdoes the latter in terms of structure predictions. The approach holds great promise for accurate and efficient RNA tertiary structure prediction.

## Author Summary

RNA is an important and versatile macromolecule participating in a variety of biological processes. In addition to experimental approaches, computational prediction of 3D structure of RNAs and loops is an alternative and important source of gaining structure information and insights into their functions. The prediction of RNA loop structures is of particular interest since RNA functions often reside in the loop regions and about 46% of nucleotides in an RNA chain remain unpaired. For this purpose, we develop an approach RNApps based on a probabilistic coarse-grained RNA model. The probabilistic nature of the model, together with a sequential Monte Carlo (SMC) growth algorithm, allows a natural and continuous sampling of structures in 3D space, making the approach unique. The coarse-graining nature of the model further increases the efficiency. Here we test this new approach with various types of loops, including hairpin loops, internal loops, and multi-way junction loops, and make comparisons with other structure predictors.

## Introduction

RNAs are a type of macromolecule of crucial and versatile biological importance. In addition to their long-discovered functions of carrying genetic information and acting as a part of translation machinery, recently they were found to be able to participate in the regulation of gene expressions and protein synthesis, and to act as scaffolds for higher-order complexes and transmit signals between cells, etc [1–4]. To fully understand the function of RNAs, knowledge of their three-dimensional (3D) structure is often required. Although the most reliable sources of RNA structural information are experimental measurements from X-ray crystallography, nuclear magnetic resonance (NMR) spectroscopy, and cryoelectron microscopy, such experiments are costly or technically challenging due to the physical chemical nature of RNAs. As a result, computational prediction of RNA structures provides a valuable alternative source of gaining structural information. Many programs have been developed to assist RNA 3D structure prediction, including YAMMP [5], NAB [6], ERNA-3D [7], MANIP [8], S2S [9], FARNA [10], MC-Fold/MC-Sym [11], RNA2D3D [12], iFoldRNA [13, 14], NAST [15], Assemble [16], HiRE-RNA [17], FARFAR [18], RNABuilder [19], ModeRNA [20], OxRNA [21], 3dRNA [22], a coarse-grained model that includes salt effect [23], our pk3D [24], etc. However, this is not a complete list due to the rapid development in the field.

The prediction of the tertiary structures of RNA loops, as part of the effort of structure prediction, merits specific attention. First, this is because RNA functions often reside in loop regions and about 46% of nucleotides in an RNA chain remain unpaired, according to Dima and her colleagues’ research [25]. Some people mistake loop regions as unstructured. Some people mistake loop regions as structured. There are stacking interactions between neighboring bases, and other non-canonical contacts. These enthalpies sometimes dominate the entropic components so that order dominates. Other times entropy dominates. Second, the prediction accuracy of the loop regions is usually lower than that of the helical regions, mostly due to the high flexibility of loops and the difficulty in calculating the energy of non-canonical base pairs and base triples frequently observed in loops. Previously, several methods have been developed for loop structure predictions. For example, Das and co-workers developed a deterministic stepwise assembly (SWA) method, and with the Rosetta statistical potential this brute-force method either reaches atomic accuracy or exposes flaws in the energy function for a testing set of 15 RNA loops [26]. Liu and Chen designed a set of dinucleotide-based statistical potentials for RNA loops and junctions and combined them with their Vfold model, then they made predictions for the coarse-grained (CG) 3D structures of both RNA loops and junctions [27]. One unique advantage of their approach is its ability to go beyond the native structures by accounting for the full free energy landscape, including all the non-native folds. Frellsen and colleagues developed a program BARNACLE (BAyesian network model of RNA using Circular distributions and maximum Likelihood Estimation) to remove some important limitations associated with the discrete nature of fragment assembly methods [28]. BARNACLE is a probabilistic RNA model based on dynamic Bayesian network and is able to efficiently sample conformations in a natural, continuous 3D space. It is shown that it captures several key features of RNA structure, and readily generates native-like conformations for 9 out of 10 test structures, solely using coarse-grained base-pairing information.

In this work we develop an approach that is specifically designed for predicting the tertiary structures of RNA loops or missing fragments which can be part in helical regions and part in loop regions. The loops can be hairpin loops, internal loops, or multi-way junction loops. The approach includes a probabilistic CG RNA model, a sequential Monte Carlo growth method, a simulated annealing strategy, and a statistical potential used for scoring the generated structural candidates. The probabilistic model is inspired by BARNACLE. However, the original BARNACLE model considers all the seven torsional angles in the RNA chain, as is not always necessary since some dihedral angles are relatively rigid. The advantage of the new model developed here is multifold. First, the probabilistic nature of the model allows a continuous sampling in the conformational space and therefore is able to cover all the relevant conformations, as is difficult for fragment assembly methods or our previous one based on a discrete-state model [24, 29]. Second, by adjusting the probabilistic functions in the model, we are able to deliberately enhance the sampling in a specific phase space. Third, the coarse-grained model further increases the efficiency, making it possible to deal with large RNAs. We name this new approach as RNApps, short for RNA structure Prediction with a Probabilistic Sampling strategy. We test this approach with a benchmark set including nine loops, a 23S ribosomal RNA, and a non-redundant RNA 3D structure dataset containing 876 RNAs [30]. We also compare the performance of our approach with other predictors.

## Methods

### The coarse-grained RNA model

**Fig 1:**
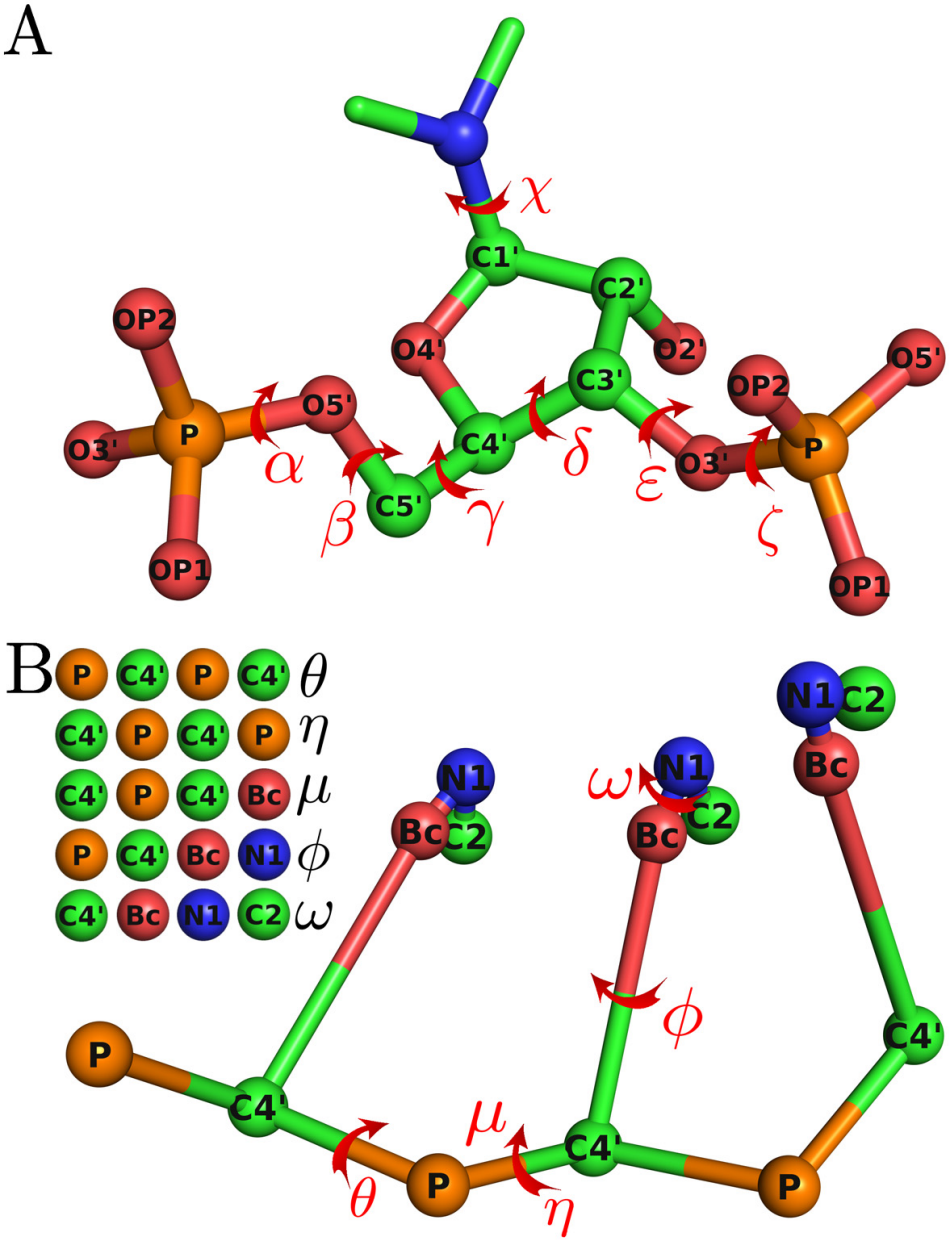
Schematic representation of all-atom and CG model for RNA. (A) Seven torsional angles of an RNA molecule. (B) Five torsional angles defined in the coarse-grained model in this work. The relevant four beads for each torsional angle are shown on the top left.

The atomistic structure of an RNA is defined by seven torsional angles, as illustrated in Fig 1A. However, the usage of such an all-atom model is expensive for structure modeling, particularly when high-throughput structure prediction is needed. In our approach, an RNA molecule is described by a CG model that includes a virtual bond model for the backbone and a reduced model for the nucleobases. The virtual bond model can be traced back to Olson [31], where the RNA backbone includes only two types of atoms, i.e., P and C4’. The model has been used in much work [32–35] and is also used here to describe the RNA backbone. For the representation of the RNA bases, three beads are used, including the atoms N1 and C2 in the purine (or pyrimidine) ring, and a virtual bead Bc (short for base center) at the geometric center of the six-membered ring containing atoms N1, C2, N3, C4, C5, and C6. In total there are five beads for each nucleotide in the CG model.

In the current model all the bond lengths and bond angles are fixed. Five torsional angles are defined based on the CG model, including *θ*, *η*, *μ*, *ϕ*, and *ω*, as illustrated in Fig 1B. Note that in the implementation there are actually two sets of such angles used for the purpose of growing RNA chains. The first set is defined in a “forward” way, i.e., from the 5’-end of the RNA to its 3’-end; and the other is defined in a “reverse” way, i.e., from the 3’-end to the 5’-end. These two sets are denoted by the subscript “+” and “−”, respectively. More specifically, from the 5’-end to the 3’-end, *θ*_+_ is calculated as the torsional angle formed by P_i_-C4’_i_-P_i+1_-C4’_i+1_, *η*_+_ by C4’_i_-P_i+1_-C4’_i+1_-P_i+2_, *μ*_+_ by C4’_i_-P_i+1_-C4’_i+1_-Bc_i+1_, *ϕ*_+_ by P_i+1_-C4’_i+1_-Bc_i+1_-N1_i+1_, and *ω*_+_ by C4’_i+1_-Bc_i+1_-N1_i+1_-C2_i+1_; from the 3’-end to the 5’-end, *θ*_-_ is calculated as the torsional angle formed by C4’_i+1_-P_i+1_-C4’_i_-P_i_, *η*_-_ by P_i+2_-C4’_i+1_-P_i+1_-C4’_i_, *μ*_-_ by C4’_i+1_-P_i+1_-C4’_i_-Bc_i_, *ϕ*_-_ by P_i+1_-C4’_i_-Bc_i_-N1_i_, and *ω*_-_ by C4’_i_-Bc_i_-N1_i_-C2_i_.

Once all the torsional angles along the RNA chain are known, the tertiary structure of the RNA molecule can be built in a “growing” way by calculating the coordinates of the to-be-grown nucleotide based on the values of the torsional angles and the previously grown nucleotides. The growing process is realized in both the “forward” way and the “reverse” way, to take advantage of the position constraints as much as possible.

### The probabilistic way of generating torsional angles

**Fig 2:**
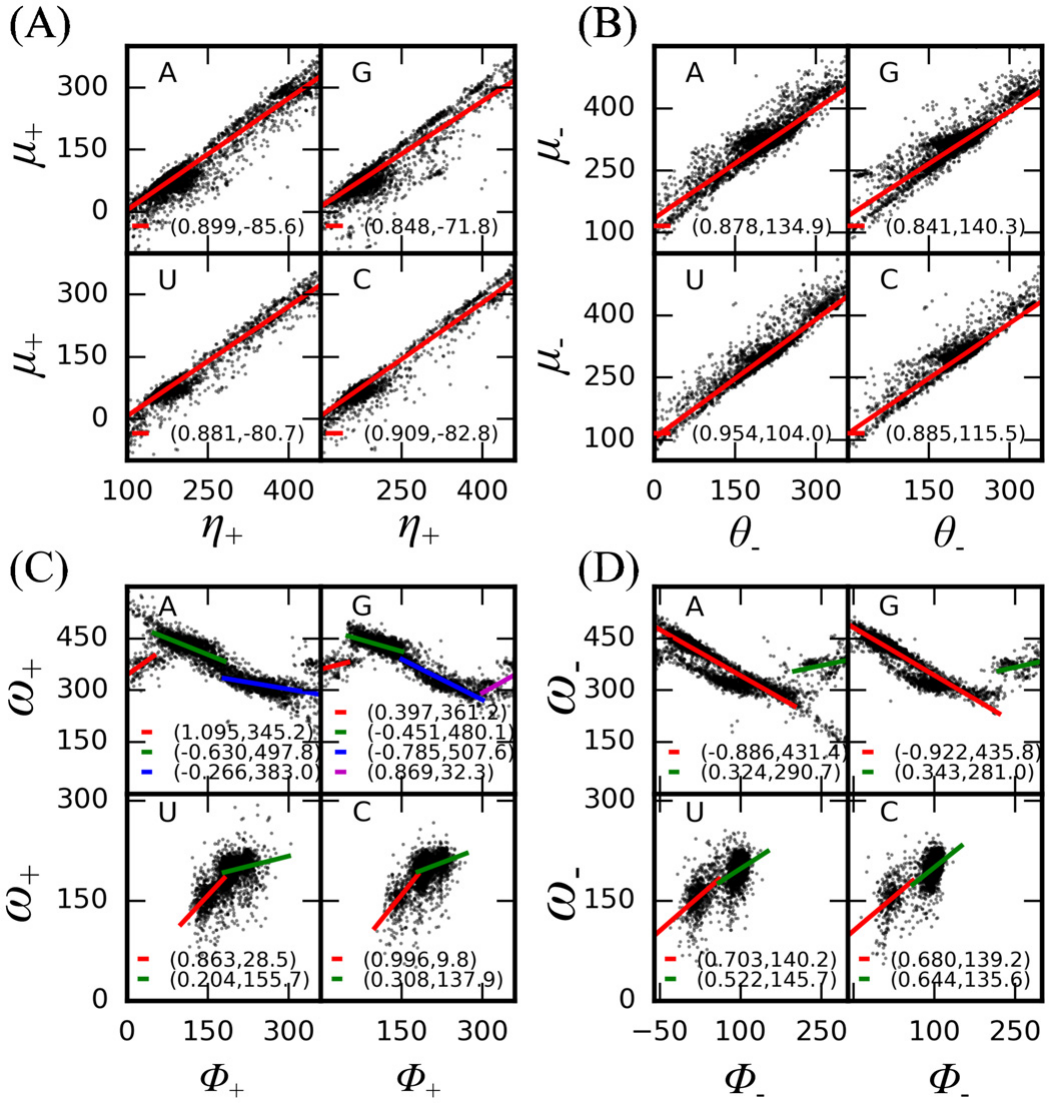
Linear relationship *y* = *kx* + *b* between (A) *η*_+_ and *μ*_+_, (B) *θ*_-_ and *μ*_-_, (C) *ϕ*_+_ and *ω*_+_, (D) *ϕ*_-_ and *ω*_-_ for four types of nucleotides. The unit is degree. Different regions in (C) and (D) show different linear correlations, indicated by the thick lines. The coefficients *k* and *b* are given in the parentheses and also listed in S1 Table for clarity. Notice: in some cases the dihedral angles are deliberately extended beyond 360° to facilitate the linear fitting process. They will be mapped back to the range [0°, 360°) during their actual usage.

**Fig 3:**
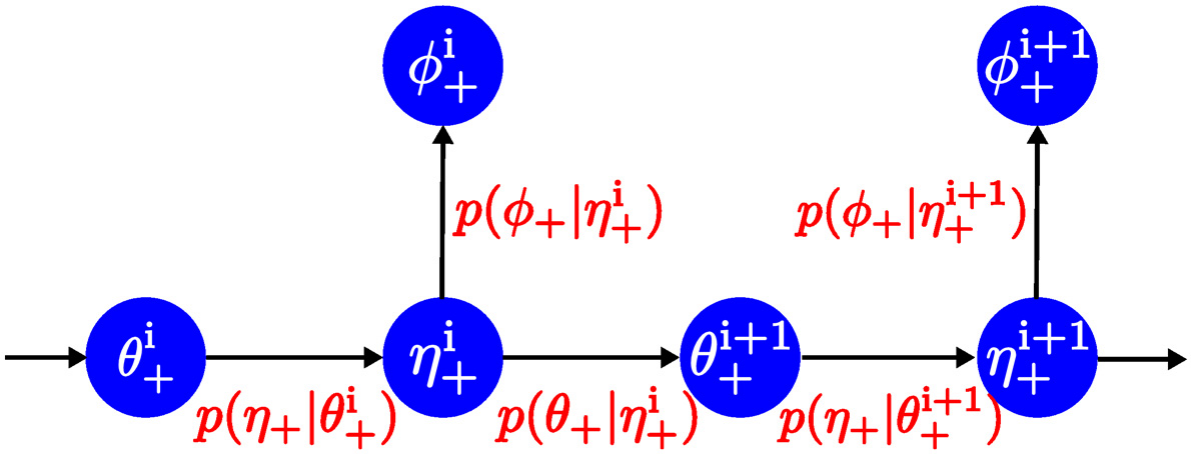
Flowchart showing how the torsional angles are determined probabilistically in a “forward” way, i.e., from the 5’-end to the 3’-end.

In the five torsional angles defined above, only three are independent, i.e., *θ*, *η*, and *ϕ*. This is because *μ* is linearly dependent on *η* (if grown in a forward direction) or *θ* (if grown reversely), and *ω* is approximately linearly dependent on *ϕ*, as supported by the statistics given in Fig 2 and S1 Table. The statistics are based on the RNA09 database compiled by Murray and co-workers by applying quality-filtering techniques (using resolution, crystallographic B factor, and all-atom steric clashes) to the backbone torsional angle distributions from a large RNA database [36]. Based on the above results the task of growing an RNA structure is reduced to determining the three torsional angles *θ*, *η*, and *ϕ*, and it is done in an iterative and probabilistic way, as illustrated in Fig 3. For example, if we are growing a loop from the 5’-end to the 3’-end, for a given $\theta_{+}^{i}$, the value of $\eta_{+}^{i}$ can be obtained probabilistically from the conditional probability $p(\eta_{+}|\theta_{+}=\theta_{+}^{i})$. Similarly, the value of $\phi_{+}^{i}$ can be calculated from the just obtained $\eta_{+}^{i}$ and the conditional probability $p(\phi_{+}|\eta_{+}=\eta_{+}^{i})$. The value of $\theta_{+}^{i+1}$ of the next nucleotide can be obtained from $\eta_{+}^{i}$ and $p(\theta_{+}|\eta_{+}=\eta_{+}^{i})$, so on and so forth. The loop may also be grown in a reverse direction, i.e., from the 3’-end to the 5’-end, and the procedure is similar but with a different set of conditional probabilities defined in a reverse way.

**Fig 4:**
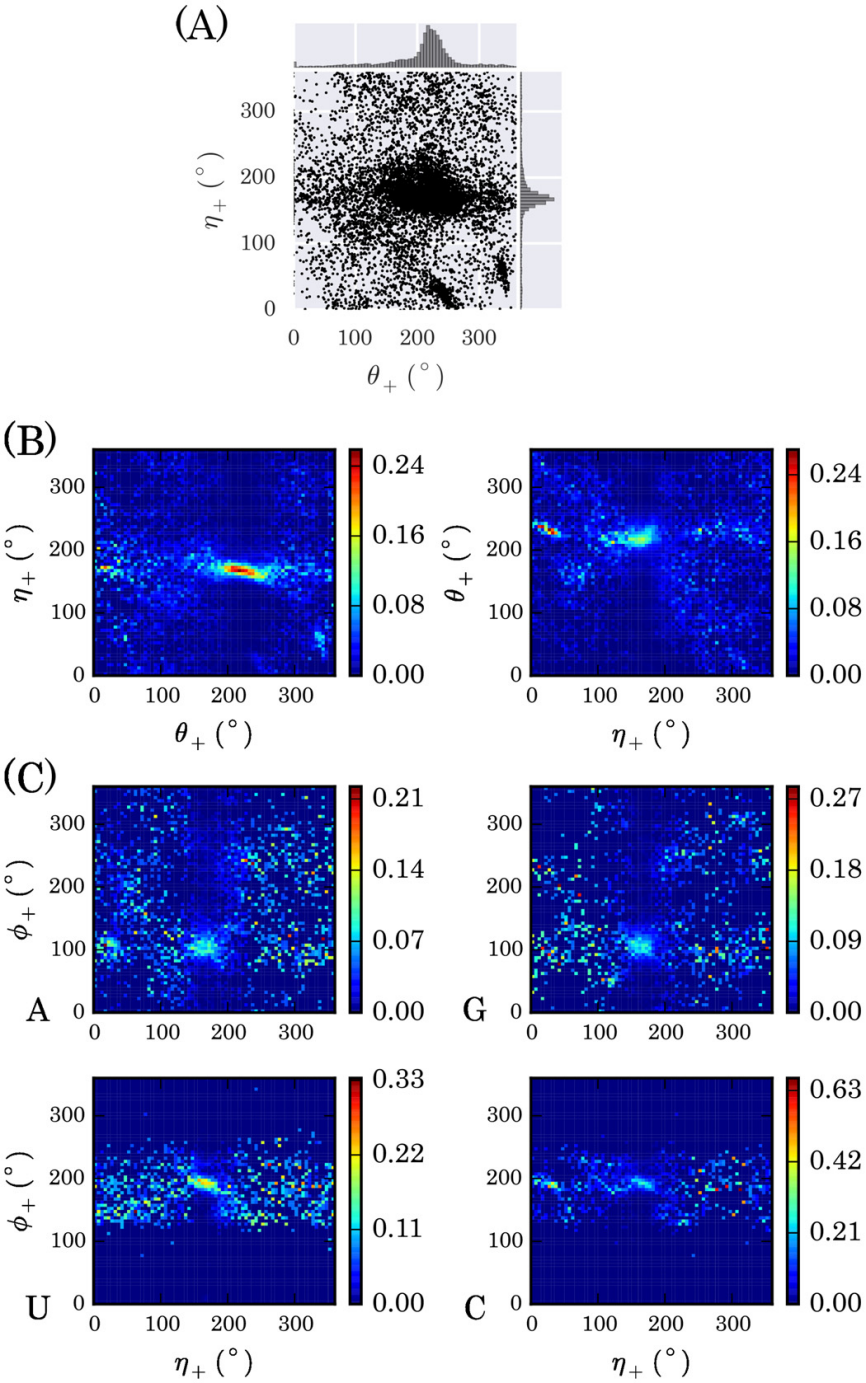
Conditional probabilities between different pairs of torsional angles. (A) Distribution of the raw data of the consecutive *θ*_+_ and *η*_+_ torsional angles in loops obtained from RNA09 database. (B) The conditional probability distribution of *p*(*η*_+_|*θ*_+_) and *p*(*θ*_+_|*η*_+_). (C) The conditional probability distribution of *p*(*ϕ*_+_|*η*_+_). Note that the distributions are dependent on the type of the nucleotides, while in *p*(*η*_+_|*θ*_+_) and *p*(*θ*_+_|*η*_+_) such dependence is ignored approximately. See the discussion in the text.

The conditional probabilities are calculated from the loop structures in the RNA09 database. Before the calculation, we first strip the helices in the structures. If two consecutive base pairs are formed, they are determined to be a helix. Three types of base pairs are considered, including A-U, G-C, and G-U. Take *p*(*η*_+_|*θ*_+_) as an example. We first calculate all the (*θ*_+_,*η*_+_) pairs in the loops in the database and plot the data points in a two-dimensional surface, as shown in Fig 4A. We then equally divide both torsional angles into *K* bins and calculate the conditional probability numerically as follows

$$p\left(\eta_{+}\left(j\right)|\theta_{+}\left(i\right)\right)=\frac{n_{ij}}{N_{i}}\;\left(i,j=1,2,\ldots ,K\right),$$ (1)

where *θ*_+_(*i*) and *η*_+_(*j*) are the center of the *i*-th and *j*-th bins, respectively, *N*_*i*_ is the number of data points with their *θ*_+_ values falling into the *i*-th bin, and *n*_*ij*_ is the number of data points with their *θ*_+_ values falling into the *i*-th bin and *η*_+_ falling into the *j*-th bin simultaneously. *K* is set to 72, and thus the bin width is Δ = 360°/*K* = 5°. The other conditional probabilities are similarly calculated. The conditional probabilities between *η*_+_ and *θ*_+_ and between *ϕ*_+_ and *η*_+_ are shown in Fig 4B and 4C, respectively. The latter are classified into four types according to the nucleotide to which *ϕ*_+_ belongs, while the former are not. This is because one (*θ*, *η*) pair involves two nucleotides and thus there are 16 types of dinucleotides; to classify the data into 16 types will lower the statistical quality since the number of data is limited.

### Structure growth with a sequential Monte Carlo method

Based on the probabilistically generated torsional angles, we build the CG structure of RNA loops/fragments by adding nucleotides one by one on the previously grown nucleotides with a sequential Monte Carlo (SMC) method [37], which is employed to prune the growing tree and bias the conformations to those having low energies and satisfying specific constraints. The constraints may include the excluded volume effect, the requirement of chain connectivity, experimental information, etc. The specific algorithm is similar to that used in our previous works [24, 29, 32, 38–41], but is improved here by growing the chain in two directions alternatively.

Specifically, for a loop sequence *X*_1_ *X*_2_⋯*X*_*n*_ whose structure is to be predicted, we grow it alternatively in two directions in 3D space from two anchor nucleotides to which this loop is attached. The algorithm also works if there is only one anchor, which happens when the loop is at one end of the RNA. To be more specific, for the *i*-th nucleotide to be grown, we randomly determine *D* = 10 possible positions of attaching it to the (*i* − 1)-th nucleotide (if grown in a forward way) by probabilistically generating the corresponding *θ*, *η*, and *ϕ* values based on the algorithm described in Fig 3 and the conditional probabilities exemplified in Fig 4. In order to increase the success rate of connecting the growing loop to the anchor nucleotide, we exclude the newly grown nucleotide whose P atom is farther from the P atom in the target anchor than a threshold, which is related to the number of nucleotides between these two nucleotides. This early pruning of the unlikely partial chain will also save a lot of computational time. After appending the *D* random configurations, we obtain a total of *N* = *L* × *D* partial chains, where *L* is the number of chains before the growth of the *i*-th nucleotide. If *N* is greater than a threshold *M*, we do a resampling procedure by randomly choosing *M* partial chains out of *N* with the probability of choosing the *m*-th one proportional to exp(−*E*_*m*_/*RT*_1_), otherwise we keep all the *N* chains. Here *E*_*m*_ is the statistical energy of the *m*-th partial chain that will be described later. The temperature *T*_1_ is used to control the relative probabilities between the candidates and *RT*_1_ is set to 300 RASP energy unit.

The above procedure works for a single chain. For internal loops or multi-way junction loops with more than one chain, the procedure is similar. Take three-way junction loop as an example. We first build *M* conformations for the first chain using the SMC method described above. Next for each in *M* conformations, we grow *D* conformations for the first nucleotide in the second chain and perform resampling to select *M* conformations out of *N* = *M* × *D* ones. We repeat these steps for the rest nucleotides in the second chain and then for the third chain. Finally we get *M* chains after the whole three-way junction loop is built.

The parameters *M* and *D* are chosen as a compromise between the accuracy and efficiency of the SMC algorithm. A large value increases the accuracy while deteriorates the efficiency, whereas a small value does the opposite. We use *M* = 80 and *D* = 20 for reasonable run time. These values are the default in this work unless otherwise stated.

### Further optimization with FRESS and simulated annealing

The growth procedure based on sequential Monte Carlo gives *M* structural candidates with low energies. These structures are further optimized with simulated annealing (SA) [42, 43]. During the SA procedure, we adopt the FRESS method (fragment regrowth via energy-guided sequential sampling) developed by Zhang et al. [44] to update the structures. In detail, for each among *M* structural candidates,

1. for a loop of length *n*, we randomly generate a positive integer *l* within *l*_max_ = min{6, *n*} with Poisson distribution *λ*^*l*^e^-*λ*^/*l*! where *λ* = 3;
2. with a uniform probability we randomly choose a nucleotide-*i* between the first and the (*n* − *l* + 1)-th nucleotide, and then delete the fragment of length *l* starting from nucleotide-*i*;
3. this fragment is regrown following the sequential Monte Carlo procedure described above, and the new fragment with the lowest energy is selected out of *M* ones as a candidate to replace the deleted one;
4. a Metropolis criterion is then used to determine whether the new fragment is accepted or not; specifically, the probability is calculated as min{1, exp[(*E*_old_ − *E*_new_)/*RT*_2_]}, where *E*_old_ and *E*_new_ are the energies of the deleted and regrown fragments, respectively; *T*_2_ = *l* * *T*_3_ is the controlling temperature proportional to the regrown loop length *l*, since the strength of interactions needed to break to escape from local minima increases with loop length; if the newly grown fragment is accepted, the new conformation of the whole loop is recorded for following cluster analysis;
5. the above procedures (i) to (iv) are repeated 50 times at each temperature *T*_3_ while *RT*_3_ is lowered linearly with cooling rate 0.8 from 100 to the lower bound 10 RASP energy unit;
6. the recorded structures during SA process are clustered using *g_cluster* command in software Gromacs with gromos cluster method [45] and cutoff 2.5 Å; the structure at the center of the largest cluster is used to evaluate the performance of the approach and make comparisons with the other methods.

### The statistical energy function

In both the SMC and SA procedures, we adopt the Ribonucleic Acids Statistical Potential (RASP) developed by Capriotti et al. [46]. In brief, RASP is an all-atom knowledge-based or statistical potential derived from a non-redundant set of 85 RNA structures. The statistical energy is calculated as a function of atom types, distance and sequence separation. It also includes a representation for local and non-local interactions in RNA structures. The threshold of sequence separation used to differentiate these two interactions is optimized with information theory. The base pairing and base stacking interactions are implicitly incorporated in the potential. More details of the RASP potential can be found in the reference [46]. The reason for choosing RASP over the other all-atom energy functions such as AMBER force field is multifold. First, it is a statistical potential and therefore more consistent with the statistical model developed here. Second, according to the authors RASP performs better than ROSETTA FARFAR force field in the selection of accurate models. Third, it is easier to be incorporated into our C++ code.

Since RASP is an all-atom statistical potential, the corresponding all-atom structures of the CG conformations generated by our approach need to be reconstructed. The procedure is as follows. Based on the atoms of the nucleobases, namely N1, C2, and Bc, the coordinates of the heavy atoms in the nucleobases are calculated with the pre-built templates taken from the RNA09 database. There are four such templates, built for A, U, G, and C, respectively. The coordinates of atoms in the backbone are calculated based on the coordinates of the atoms P, C4’, and N1 (in the bases of U and C) or N9 (in the bases of A and G) with a pre-built template. After the reconstruction we check if there are steric clashes between atoms. If any clash is found, the structure is discarded.

## Results

### Testing performance with a nine-RNA dataset

Here we test our approach and compare it with two other RNA tertiary structure predictors, i.e., RLooM [47] and iFoldRNA [13, 14], and also with RNAnbds [29], which was developed previously in our lab. RLooM is based on homology modeling and utilizes template structures extracted from a PDB database. iFoldRNA represents an *ab initio* way of prediction based on physical interactions and discrete molecular dynamics (MD) simulation. These two are selected to represent two very different strategies of structure prediction in the field. The testing set that appears in the original RLooM paper [47] is used for the comparison. This set contains 9 RNA loops with their length ranging from 6 to 17-nt. For RLooM, the RMSD values of the predicted loops with respect to the experimental ones were directly taken from the RLooM paper [47]. For iFoldRNA, they were obtained by feeding to the iFoldRNA-v2 server the whole sequences plus the corresponding base pairing information as constraints [14]. For RNApps, the input includes the whole sequence and the structure other than the loops. The output is the center of the largest cluster calculated from the trajectory of the SA simulation, as described in the Methods section. For a fair comparison, the RMSDs of the reduced backbones (P, O5’, C5’, C4’, C3’, and O3’ atoms) given optimal superposition with the experimental ones are calculated, following the same methodology as in RLooM and iFoldRNA. For the comparison with our previous RNAnbds, the RMSDs of all heavy atoms are used. The results are summarized in Table 1.

**Table 1:** Compare the performance of four methods with nine RNA loops.

PDB ID	1EVV	1EVV	1EVV	1L2X	1Q8N	1Q9A	1RMN	2CKY	2F88
loop	13–22	30–40	53–61	7–14	14–19	6–22	16–21	26–36	23–27
RLooM^a^	0.98	0.69	0.90	0.75	2.05	1.05	1.71	0.38	1.34
iFoldRNA^a^	3.7	3.95	4.58	4.1	1.72	6.04	1.66	4.01	1.83
RNApps^a^	3.01	0.89	1.74	2.46	0.84	7.73	1.00	6.50	1.54
RNAnbds^b^	5.6	3.5	4.8	3.7	2.0	7.6	1.7	5.6	3.0
RNApps^b^	4.69	1.27	3.30	4.51	1.54	7.33	1.32	7.92	2.26
RNApps^c^	5.28	1.84	3.80	4.67	1.92	8.63	1.39	10.68	2.37

^a^ RMSD of reduced backbone trace after optimal superposition with the experimental RNA loop.

^b^ RMSD of heavy atoms after optimal superposition with the experimental loop.

^c^ RMSD of heavy atoms after optimal superposition of the anchors. The unit is angstrom.

In the first part of the table, RNApps is compared with RLooM and iFoldRNA. It can be seen that for this specific testing set, RNApps performs comparably with RLooM or slightly worse in a few cases. This is normal since the homology modeling based predictors usually perform better than those based on energy rules. However, such predictors are sensitive to the testing set. For example, we tested RLooM with 28 fragments of length of 8-nt taken from the RNA pseudoknot 1E95 and found that RLooM failed for all of them. The reason is that there are no homological partners of this RNA in the RLooM database. In contrast, our method guarantees to give a reasonable prediction (data shown in S1B Fig). The comparison with iFoldRNA shows that RNApps gives smaller RMSDs in seven out of nine RNAs, and slightly larger RMSDs in two cases. Therefore, for this testing set taken from the third party (the RLooM paper), our method shows a better performance than iFoldRNA. However, it is also worth mentioning that iFoldRNA is not only a structure predictor, but also can be used for studying folding dynamics as a result of the employed molecular dynamics simulation.

We then compare RNApps with our previous RNAnbds. It can be seen that the new approach has a considerably better performance, giving smaller RMSDs for seven out of nine loops and two larger RMSDs. The improvement is mostly attributed to the feature that RNApps is able to sample the structural space in a continuous way, while RNAnbds is based on a discrete-state RNA model and thus unable to reach some structural regions. The different energy functions employed may also contribute. In Table 1 we also give the RMSDs of heavy atoms calculated after optimal superposition of the anchors—the predicted and the experimental loops themselves are not superimposed. These RMSDs characterize how well the relative positions of the loops with respect to their environment are reproduced. It can be seen that the RMSDs are only slightly larger than those calculated after optimal superposition of the loops, indicating that this performance is good.

**Fig 5:**
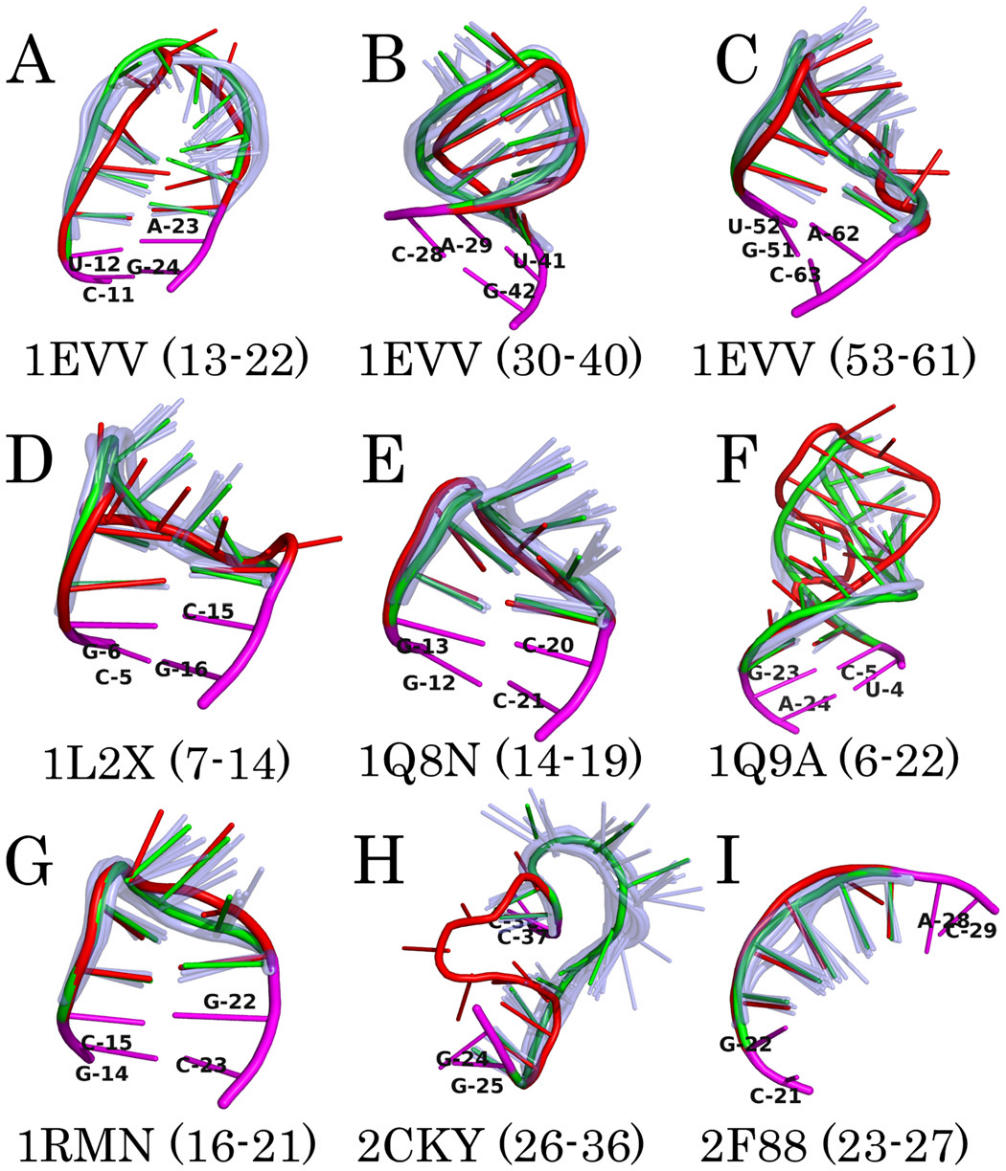
Superposition of the predicted and the experimental structures of nine loops, shown in the same order as in Table 1. The red cartoons represent the experimental conformations. For each loop, the light-blue and semi-transparent cartoons are the first 20 structures in the largest cluster, whose central structure is shown by the green cartoon. The magenta cartoons mark the anchor nucleotides.

In Fig 5 we superimpose the predicted structures with the experimental ones for the nine RNAs to make a visual comparison. Since the approach actually predicts a structural ensemble, for each sequence we also show the largest cluster to which the predicted structure belongs. It can be seen that for seven out of nine loops, the predicted structures are similar to their corresponding experimental ones. As for the structural feature of the cluster, the trends of the backbones correlate well with each other, while the bases are somehow dynamic if they do not form base pairs.

The loops in 1Q9A and 2CKY (Fig 5F and 5H) are exceptional. In 1Q9A the concerned loop is known as the bulged-G motif, which is a ubiquitous loop and provides specific recognition sites for proteins and RNAs [48]. More specifically, it is an essential part of the binding site for elongation factors in *Escherichia coli* 23S ribosomal RNA. In this motif, one strand forms a kink so that the bulged-G can form a base triple by non-canonical interactions, permitting interstrand, but disrupting intrastrand stacking. The kink in one strand, together with the S-turn in the opposite strand, appears to make the motif amphipathic, with one surface more polar and ready for interacting with positive patches on proteins or a divalent metal ion, and the other surface less polar and suitable for making RNA-RNA contacts [48]. The above features indicate that this motif is highly optimized for binding its partners, which should be considered when making structure predictions. The RNA 2CKY, which is a riboswitch specifically recognizing the thiamine pyrophosphate, has a similar situation [49]. The loop shown in Fig 5H is in a highly distorted region that binds its ligand. These results suggest that, the consideration of the binding partners, if they have, is necessary for a correct prediction of the tertiary structures of RNAs.

### Testing performance with a ribosomal RNA

**Fig 6:**
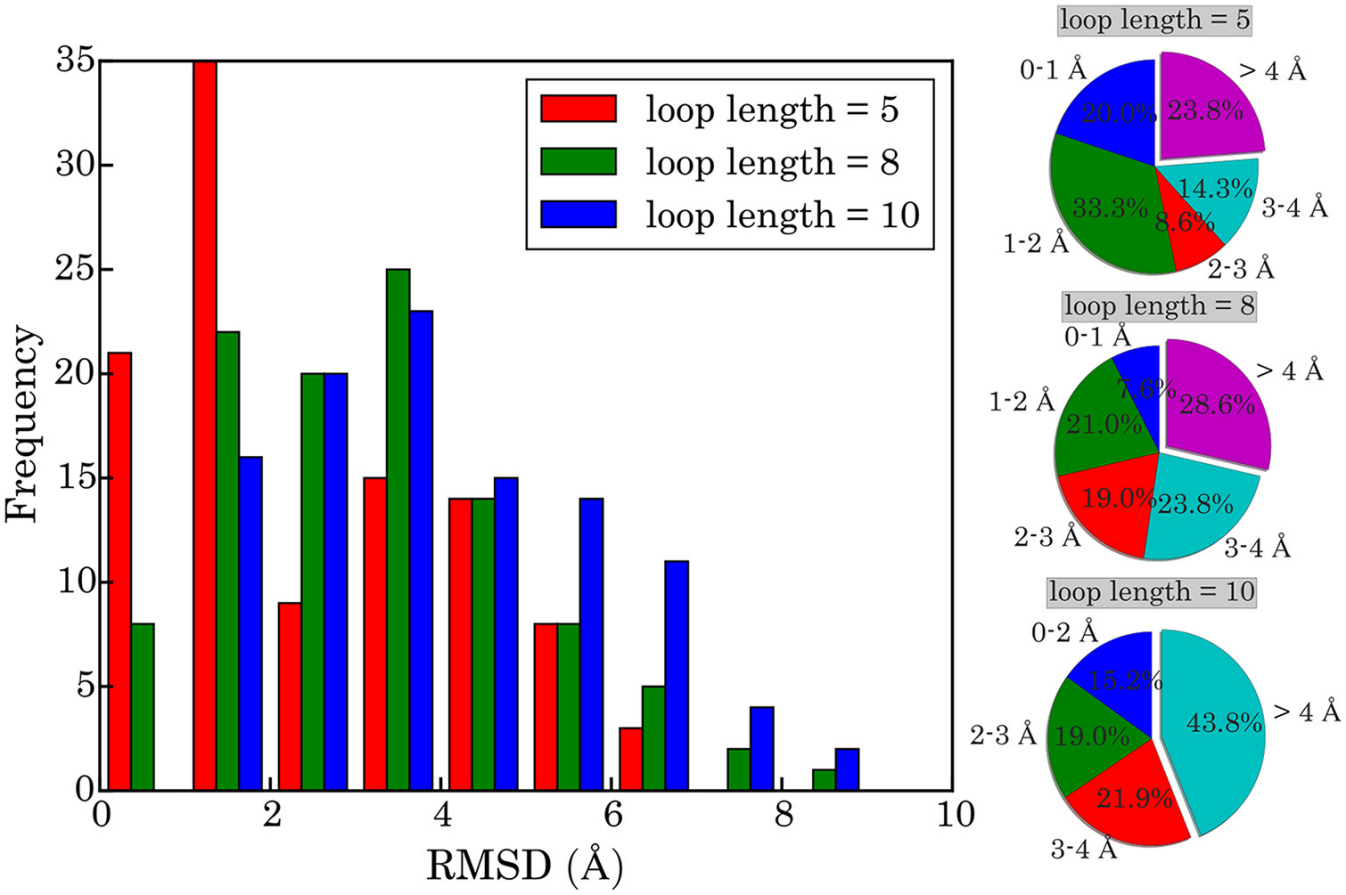
Distribution of RMSDs of 105 fragments in 1FFK of different length (5-, 8-, and 10-nt). For each fragment, the central structure of the largest cluster was taken as the predicted structure. The RMSDs are calculated based on heavy atoms after optimal superposition of the predicted structures with the experimental ones. The pie charts show the proportions of RMSDs within certain ranges indicated by the labels; from top down they correspond to the fragments of length 5, 8, and 10, respectively.

Our approach can also be used for reconstructing a missing RNA fragment, in addition to a single loop. The fragment can be a mixing of helical and loop regions. Here we show its performance with a 23S ribosomal RNA (PDB ID 1FFK) [50] consisting of about 3,000 nucleotides. In total we make 105 tests, and in each test we delete one fragment of length *N* and then reconstruct its tertiary structure, pretending that the fragment is missing in the RNA structure. The fragments are chosen every *S* nucleotides along the sequence, where *S* is set to 25 so that the fragments are uniformly distributed in the whole RNA. The length *N* can be 5, 8, and 10, and the percentage of nucleotides in the helical region in the 105 fragments is 36%, 39%, and 39%, respectively. According to Fig 6, the percentage of RMSDs smaller than 4 Å is 76.2%, 71.4%, and 56.2% for the fragments of length 5, 8, and 10, respectively. The average RMSDs in the three cases are 2.55 Å, 3.23 Å, and 3.97 Å, respectively.

### Further testing with a large dataset

We further test our approach with a much larger dataset—RNA 3D Hub [30]. It contains 876 non-redundant RNAs (release ID 1.89), including all types of base-pairing, base-stacking, and base-backbone interactions. Out of this dataset we select 1768 hairpin loops, 1649 internal loops, 230 three-way junction loops and 113 four-way junction loops. We then delete these loops from the molecules and reconstruct them following the procedure described in the Methods section. For each loop, the structure at the center of the largest cluster is taken as the predicted structure.

**Fig 7:**
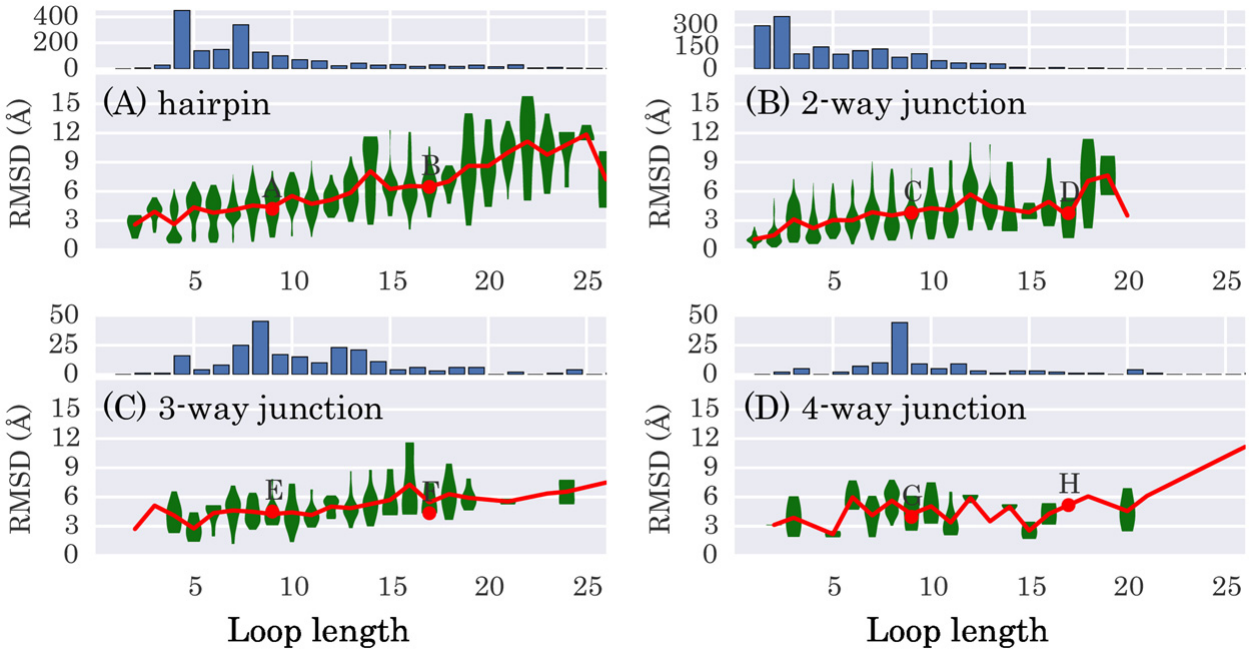
Violin plot of the RMSDs as a function of loop length for four types of loops in the dataset RNA 3D Hub. The red lines denote the mean RMSDs between predicted structures and experimental structures. The green body of each violin represents a kernel density estimation of the RMSD distribution. The histogram on the top of each figure gives the loop length distribution in the dataset. Figures (A), (B), (C), and (D) correspond to 1768 hairpin loops, 1649 internal loops, 230 three-way and 113 four-way junction loops, respectively. RMSDs are calculated based on heavy atoms after optimal superposition of the predicted structures with the experimental ones. The loops exemplified in Fig 8 are drawn as red dots and labeled from A to H.

In Fig 7 we show the RMSDs of the predicted structures with respect to the experimental ones as a function of loop length. Overall, for hairpin loops, internal loops, and three-way junction loops, the RMSDs increase gradually as the loop length increases. The average RMSDs are around 4 Å for hairpin loops of length smaller than 10-nt and increase from 5 to 9 Å for loops of length from 10- to 20-nt. For even longer loops, the average RMSDs are around 11 Å. For internal and three-way junction loops, the performance is better than that for hairpin loops, as may be attributed to the increased number of anchor nucleotides in these cases. For four-way junction loops, the average RMSDs undergo large fluctuations, due to the relatively smaller number of such loops in the dataset. The average RMSDs generally range from 3 Å to 6 Å for loops of length smaller than 20-nt.

**Fig 8:**
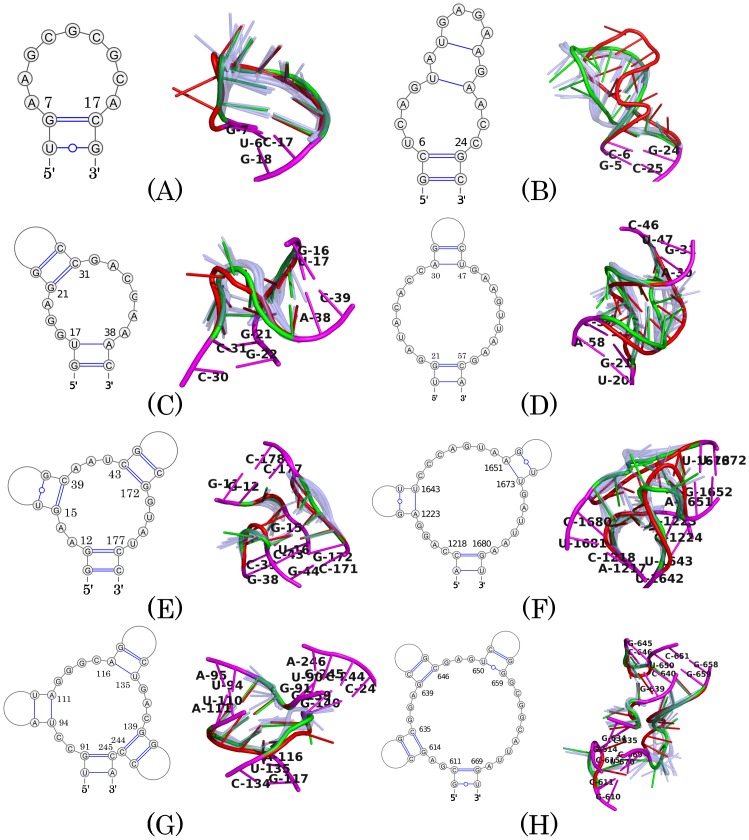
Superposition of the predicted and the experimental structures for four types of loops. The experimental structures are colored red, the predicted loops are green, and the first 20 structures in the largest cluster are colored light-blue and semi-transparent. The anchor nucleotides are colored magenta. (A) to (H) correspond to the data points of the same labels shown in Fig 7. (A)(B) are hairpin loops, (C)(D) internal loops, (E)(F) three-way junction loops, (G)(H) four-way junction loops. The loop length and RMSD are as follows: (A) 9 nt and 4.21 Å, (B) 17 nt and 6.46 Å, (C) 9 nt and 3.81 Å, (D) 17 nt and 3.76 Å, (E) 9 nt and 4.51 Å, (F) 17 nt and 4.34 Å, (G) 9 nt and 3.96 Å, (H) 17 nt and 5.17 Å.

In Fig 8 we show two examples for each one of four cases for visual comparison—one example has a medium loop length (9-nt) and one has a long loop length (17-nt). The loops with their RMSDs close to the average (marked by the red dots near the red lines in Fig 7) are selected, so that they are most representative. For the eight cases shown here, six loops are predicted with a reasonable accuracy, with RMSDs around 4 Å. Visual inspection of the figures shows that, the backbones of the predicted structures match those of the experimental ones, while the bases are somehow dynamic (e.g., Fig 8A and 8C).

Two loops are less well predicted. The loop shown in Fig 8B is an essential part of the binding site for elongation factors in rat 28S rRNA. The RMSD between the predicted structure and the experimental one is 6.46 Å. This relatively large value is due to the failure of reconstructing the bulge-G motif, similar to the case discussed in Fig 5F.

The loop of length 17-nt shown in Fig 8H belongs to a four-way junction, locating at the surface of a large ribosomal RNA. The loop is formed by four sub-loops of length 2-, 3-, 3-, and 9-nt, respectively. The RMSD between the predicted structure and the experimental one is 5.17 Å. In the experimental structure, the longest sub-loop forms little interaction with the other three ones and extends out into the solvent. The prediction well reproduced a conformation with this kind of characteristics. The largest deviation between prediction and experiment is in the 2-nt sub-loop G612-A613 and 3-nt sub-loop G647-A648-G649, where A613 and A648 are both in a sharp turn and form a stacking interaction with A668 and A638, respectively.

## Discussion

In this work we develop an approach named RNApps specifically designed for loop structure prediction. The approach includes a probabilistic coarse-grained RNA model, a sequential Monte Carlo growth algorithm, a simulated annealing procedure and an all-atom statistical energy function.

We tested the approach with a set of nine RNA loops, a 23S ribosomal RNA, and a large RNA dataset containing 876 RNAs (RNA 3D Hub, release ID 1.89). For the testing set including nine RNA loops, six loops can be predicted with good accuracy (RMSD < 2.5 Å), one loop has an RMSD of 3.01 Å and two have RMSDs around 6-7 Å. We compared the results with a homology modeling based predictor RLooM and an *ab initio* predictor iFoldRNA. It was found that RNApps performs comparably with RLooM while considerably better than iFoldRNA. However, we also noted that RLooM cannot guarantee a return of valid structures for some targets, due to the lack of their homology information in the database. In contrast, RNApps and iFoldRNA guarantee a result, and iFoldRNA can also be used for studying the folding dynamics. The tests with a ribosomal RNA showed that the average RMSD is 2.55 Å, 3.23 Å, and 3.97 Å for the rebuilt fragments of length 5-, 8-, and 10-nt, respectively. The tests with RNA 3D Hub showed that the average RMSDs for hairpin loops are around 4 Å and increase slightly with the loop length. The performance for internal loops, three-way and four-way junction loops is even better than that for hairpin loops, mostly due to the increased number of anchor nucleotides. Further analysis showed that although most RNA loops are predicted with good accuracy, some ones with non-canonical base pairs, base triples, or rare torsional angles are reproduced with a lower quality.

We note that our approach in it current form does not perform considerably better than the other predictors and even slightly worse than the homology modeling based one. However, our approach has many unique and promising features. First, it is not only designed for predicting structures of hairpin loops, but also for internal loops, three-way and four-way junction loops and even complex cases. One motivation is that many predictors have been designed to predict the relative position and orientation of the helices of an RNA molecule, however, less attention was paid to the construction of the loops connecting the helices. This makes our work necessary. Second, the efficiency of our approach is high. With the parameters used in this work, a prediction of loop with medium length takes several minutes without SA optimization or several tens of minutes with SA optimization. For example, the computational time for the longest loop (26-nt) in three- and four-way junctions is approximately one hour. This efficiency is much higher than that of most MD based algorithms and the approach is free from the problems associated with homology modeling based methods. The efficiency will be further improved by optimizing the SMC and SA algorithms. Third, the probabilistic and continuous nature of the approach guarantees the sampling of all the relevant phase space in principle, and allows a dynamic adjustment between accuracy and efficiency, which can be determined by users based on their own computational capacity. Fourth, the SMC framework of the approach makes the incorporation of constraints very easy. The constraints may be the experimental information of atomic distance, base pairs or base stacking, or information from users’ experience. With the introducing of such information, the sampling space can be greatly reduced and both accuracy and efficiency will be significantly improved. The way of incorporation of constraints into the SMC framework can be found in previous work [41]. We believe our approach is useful for predicting the tertiary structure of RNA loops.

We also noticed that there are two recent works in protein loop predictions that are similar to ours. In the first one, Tang et al. developed an approach named DiSGro based on sequential Monte Carlo method [51], which is the same as ours. With this approach, they are able to efficiently generate high quality protein loop conformations. The average minimum global backbone RMSD for 1,000 conformations of 12-residue loops is 1.53 Å, with a lowest energy RMSD of 2.99 Å, and an average ensemble RMSD of 5.23 Å. In the second work, the same authors upgraded their approach for the prediction of conformations of multiple interacting loops in proteins [52]. For the most challenging target proteins with four loops, the average RMSD of the lowest energy conformations is 2.3 Å. One novel feature of the approach is the simultaneous construction of multiple loops, making it less likely to over-sample conformations in certain local energy minima. This idea is naturally compatible with the framework of our approach and can be easily incorporated into it. We believe their approaches and ours can borrow ideas from each other and then improve the performance of both.

## Supporting Information

S1 Fig Tertiary structure prediction for fragments in the RNA pseudoknot 1E95. (A) Secondary structure of pseudoknot 1E95. (B) shows the RMSDs of predicted structures for all fragments of length 5, 8, and 10 in 1E95. The x-axis stands for the index of the first nucleotide of the tested fragment.
(EPS)

S2 Fig To estimate as to what percentage of genomic loop regions have no sequence homologs to structural templates, we calculated a quantity termed similarity probability for loops in the nine-loop testing set. For each loop (denotes as *p*) in the nine-loop testing set, the quantity termed similarity probability is defined as the number of (*θ*,*η*) pairs in the loops of the same sequence as *p* in the templates over the total number of (*θ*,*η*) pairs in all the loops in the templates. As shown in the figure, the similarity probabilities are all less than 0.5%. Therefore, the presence of homologs has minimal effect on the conditional probability and hence the final prediction results.
(EPS)

S1 Table Values of coefficients *k* and *b* in Fig 2, the corresponding RMSE (root mean squared error) and domains. (PDF)
