# Supplementary material for: Structure Prediction of RNA Loops with a Probabilistic Approach
Source: PLoS Comput Biol. 2016 Aug 5;12(8):e1005032. doi: 10.1371/journal.pcbi.1005032 (PMC4975501; doi:10.1371/journal.pcbi.1005032)
Supplement: S1 Table — (PDF) [file pcbi.1005032.s003.pdf]

S1 Table. Values of coefficients k and b in Fig. 2, the corresponding RMSE and domains.

	A	G	U	C
η_+-μ_+	$\mathbf{(0.899, -85.6)}$<br>RMSE=22.6°<br>$100° < \eta_+ < 460°$	$\mathbf{(0.848, -71.8)}$<br>RMSE=20.7°<br>$100° < \eta_+ < 460°$	$\mathbf{(0.881, -80.7)}$<br>RMSE=16.7°<br>$100° < \eta_+ < 460°$	$\mathbf{(0.909, -82.8)}$<br>RMSE=14.4°<br>$100° < \eta_+ < 460°$
θ_--μ_-	$\mathbf{(0.878, 134.9)}$<br>RMSE=22.2°<br>$0° < \theta_- < 360°$	$\mathbf{(0.841, 140.3)}$<br>RMSE=19.3°<br>$0° < \theta_- < 360°$	$\mathbf{(0.954, 104.0)}$<br>RMSE=20.5°<br>$0° < \theta_- < 360°$	$\mathbf{(0.885, 115.5)}$<br>RMSE=16.8°<br>$0° < \theta_- < 360°$
ϕ_+-ω_+	$\mathbf{(1.095, 345.2)}$<br>RMSE=24.5°<br>$0° < \phi_+ < 50°$<br>$\mathbf{(-0.630, 497.8)}$<br>RMSE=16.4°<br>$50° < \phi_+ < 180°$<br>$\mathbf{(-0.266, 383.0)}$<br>RMSE=29.6°<br>$180° < \phi_+ < 360°$	$\mathbf{(0.397, 361.2)}$<br>RMSE=24.6°<br>$0° < \phi_+ < 50°$<br>$\mathbf{(-0.451, 480.1)}$<br>RMSE=11.9°<br>$50° < \phi_+ < 150°$<br>$\mathbf{(-0.785, 507.6)}$<br>RMSE=20.3°<br>$150° < \phi_+ < 300°$<br>$\mathbf{(0.869, 32.3)}$<br>RMSE=34.3°<br>$300° < \phi_+ < 360°$	$\mathbf{(0.863, 28.5)}$<br>RMSE=23.0°<br>$100° < \phi_+ < 180°$<br>$\mathbf{(0.204, 155.7)}$<br>RMSE=14.3°<br>$180° < \phi_+ < 300°$	$\mathbf{(0.996, 9.8)}$<br>RMSE=24.6°<br>$100° < \phi_+ < 180°$<br>$\mathbf{(0.308, 137.9)}$<br>RMSE=11.4°<br>$180° < \phi_+ < 270°$
ϕ_--ω_-	$\mathbf{(-0.886, 431.4)}$<br>RMSE=22.8°<br>$-60° < \phi_- < 200°$<br>$\mathbf{(0.324, 290.7)}$<br>RMSE=25.7°<br>$200° < \phi_- < 300°$	$\mathbf{(-0.922, 435.8)}$<br>RMSE=15.5°<br>$-60° < \phi_- < 220°$<br>$\mathbf{(0.343, 281.0)}$<br>RMSE=23.7°<br>$220° < \phi_- < 300°$	$\mathbf{(0.703, 140.2)}$<br>RMSE=24.5°<br>$-60° < \phi_- < 60°$<br>$\mathbf{(0.522, 145.7)}$<br>RMSE=13.3°<br>$60° < \phi_- < 150°$	$\mathbf{(0.680, 139.2)}$<br>RMSE=27.3°<br>$-60° < \phi_- < 60°$<br>$\mathbf{(0.644, 135.6)}$<br>RMSE=10.8°<br>$60° < \phi_- < 150°$
